# STEB: A secure service trading ecosystem based on blockchain

**DOI:** 10.1371/journal.pone.0267914

**Published:** 2022-06-03

**Authors:** Wei Liu, Wenlong Feng, Mengxing Huang, Yun Xu, Xiandong Zheng

**Affiliations:** 1 School of Information and Communication Engineering, Hainan University, Haikou, Hainan Province, P. R. China; 2 College of Biomedical Information and Engineering, Hainan Medical University, Haikou, P. R. China; 3 Wenchang Satellite Launch Center, Wenchang, P. R. China; University College of Engineering Tindivanam, INDIA

## Abstract

A service can be an intangible commodity in which no physical goods are transferred from the seller to the buyer. However, traditional trading platforms have many limitations in trading services due to dishonest buyers and brokers. In this paper, we propose a service trading ecosystem based on blockchain, named STEB, which combines blockchain, smart contract, encryption, and digital authentication techniques for service trading. In addition, a dual-chain architecture, which contains two types of blockchains, namely TraChain and SerChain, and a hierarchical encryption scheme of the data on the chain, are proposed to ensure the integrity of transaction data and fine-grained privacy protection of users. Furthermore, we describe a new set of smart contracts to ensure safe transactions for the entire service trading. Security analysis and simulation results confirm that the proposed STEB can achieve more efficient contract execution and enhance service transaction privacy.

## 1 Introduction

Technological progress and the accelerated growth of globalization have triggered consumer demand, leading to the rapid expansion of the complexity of the trade network. Service transaction [1] is the basic characteristic of modern service industry. Service-oriented, that is, physical, hardware, and software assets are transformed into abstract virtual services, in which users establish on-demand interactions, binding resources and operations. People can not only buy traditional real goods online, but also buy virtual services. For example, a service consumer chooses a service provider, hires it to handle the application for an invention patent, and pays the fee after the application is successful. Virtual services can be traded as commodities. With the rapid growth and widespread adoption of online services, our world has accumulated a lot of resources. Resources are created, discovered, linked, analyzed, and synthesized by billions of users distributed on the Internet, thereby generating new great value. Online service transactions break and eliminate the time and space limitations of traditional transactions, making it possible at anytime, at anywhere, and with anybody.

However, one of the major challenges faced by service transaction is the lack of trust among sellers and buyers. Most of the existing trust schemes require a trusted third party to handle the transaction process. Blockchain technology, a decentralized public ledger to record transactions, has recently attracted much interest in the general public. In 2008, Nakamoto [2] proposed a distributed ledger named blockchain originated from Bitcoin. With the rise of digital currencies such as Bitcoin, blockchain has become a new technology that is decentralized, secure, credible, non-tamperable, and traceable. The consensus mechanism [3] is used to complete the verification and confirmation of the transaction in a short time. Asymmetric encryption and chain structure [4] are used for encryption and decryption and to prevent tampering with the data in the block. Blockchain has now developed into a trustworthy decentralized platform, and has been widely used in the fields of finance, supply chain, data sharing [5], energy, healthcare [6, 7], vehicular ad hoc networks [8, 9], intellectual property protection [10–12], the Internet of Things [13], and other fields [14].

In our previous study [15], we established an optimization of PBFT algorithm bsed on improved C4.5. On the basis of the previous research, we will continue to invest in the blockchain method to help the development of service transactions. We propose STEB, a decentralized service transaction system, implemented through consensus algorithms, encryption algorithms, and smart contracts on the blockchain. We aim to create a distributed, credible and secure trading environment for multi-party participants in service transactions. The main contributions of this paper can be summarized as follows:

1. We propose a novelty secure blockchain-based service trading ecosystem, named STEB, and a new set of smart contracts, to enforce consensus among multi-participants safely without trusted third parties.
2. We carefully design a dual-chain structure to be executed by smart contracts, different access rights are granted to data on the dual-chain to ensure both transaction data integrity and security.
3. We represent a hierarchical encryption scheme of the data on the chain to achieve fine-grained privacy protection of users.

We verify the feasibility of the proposed STEB by implementing it on the Hyperledger Fabric network. Furthermore, the standard tool Hyperledger Caliper is used to illustrate the effectiveness and robustness of the proposed solution.

## 2 System model

**Fig 1:**
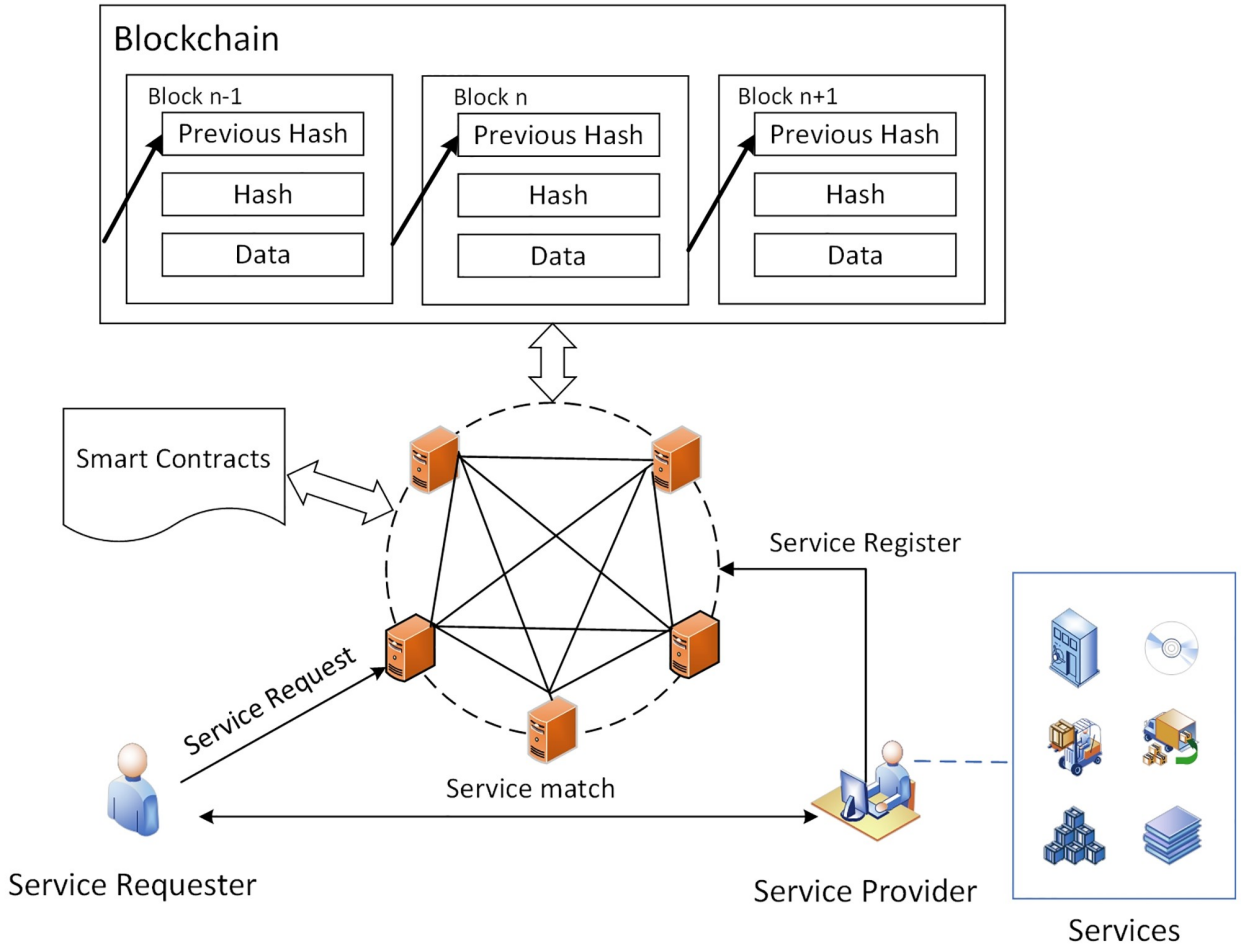
The structure of a typical system model.

As shown in Fig 1, STEB is a secure blockchain used for contract deployment, demand matching, consensus, and contract execution. Its main function is to ensure service transactions are carried out in a safe and reliable manner.

### 2.1 Nodes

A user can be a Service Requester (SR), a Service Provider (SP), or both. SP is a service provider, registering for services, providing services for SR, and profiting from selling services.

#### 2.1.1 SR node

Each one *SR*_*i*_ is an end user who can access one or more services from the SP via STEB. A *SR*_*i*_ uese one or more service request contracts, analyzes SP and its services, matches eligible services, and obtains data analysis results. When he receives feedback from STEB, he purchases services according to individual requirements. SR nodes only can read the information on SerChain, which are defined below.

#### 2.1.2 SP node

Each one *SP*_*j*_ should be authorized by STEB. The *SP*_*j*_ can be human (e.g., individual software developer) or organization (e.g., company, research institute, or inspection organization). A *SP*_*j*_ configures to register one or more services on the STEB. When the service is certified, *SP*_*j*_ offers the services for sale. All *SP*_*j*_ nodes form a consortium, and participate in consensus. The behavior of all SP nodes is restricted by the rules of the consortium. Authorized SP nodes can read the information on TraChain and SerChain, which is defined below, and generate transactions to chains.

### 2.2 Blockchain

There are two chains in STEB.

#### 2.2.1 TraChain

It is a consortium blockchain. All SPs have to be authorized via STEB and form a consortium. Only SP nodes authorized by consortium can generate services as goods. SP joins TraChain to register services, provide services, read transactions, send transactions, and mine at any time. TraChain consists of a series of SPs, their service data, and their transaction records, and grows over time. Each block contains the hash of the previous block and services as goods generated by SPs.

#### 2.2.2 SerChain

It is a consortium blockchain, which is used to publish transactions data. However, anyone can read the information on SerChain. SerChain consists of a series of service transactions blocks and grows over time. Each block contains the hash of the previous block and transactions generated by users.

### 2.3 Smart contracts

In the proposed system, three smart contracts, which are presented as Service Information Contract (SIC), SR Requirement Contract (SRC), and Service trading Management Contract (SMC) respectively, are used to manage services and transactions between users.

#### 2.3.1 SIC

The main function of the smart contract SIC is to store and manage service information, which is used to register SP users and their services in the system. To achieve this function, SIC maintains a query table. This table registers the information required to find and execute all SPs and their services. It also stores the SR’s comments of the service after each service transaction is completed, and the transaction execution information (name, attributes, etc.). The query module helps potential buyers match suitable services and appropriate sellers based on the contract information in the SIC.

#### 2.3.2 SRC

The main function of the smart contract SRC in the system is to receive and broadcast SR service demands. Whenever the SR requires any service, it will send a request to the system. The system receives SR demands and broadcasts them to SP nodes, which enables SP to know SR requirements. Every time SR generates a request, it deploys an SRC in response to the request. When the SR sends a service request to the system, the SRC is executed.

#### 2.3.3 SMC

The main function of the smart contract SMC in the system is to manage service trading. SMC records SP’s service specifications, price, and trading states, and it also provides a query module for SR to check their service trading statement based on the contract information through trade ID.

## 3 Secure service trading ecosystem based on blockchain

### 3.1 Overview of STEB

**Fig 2:**
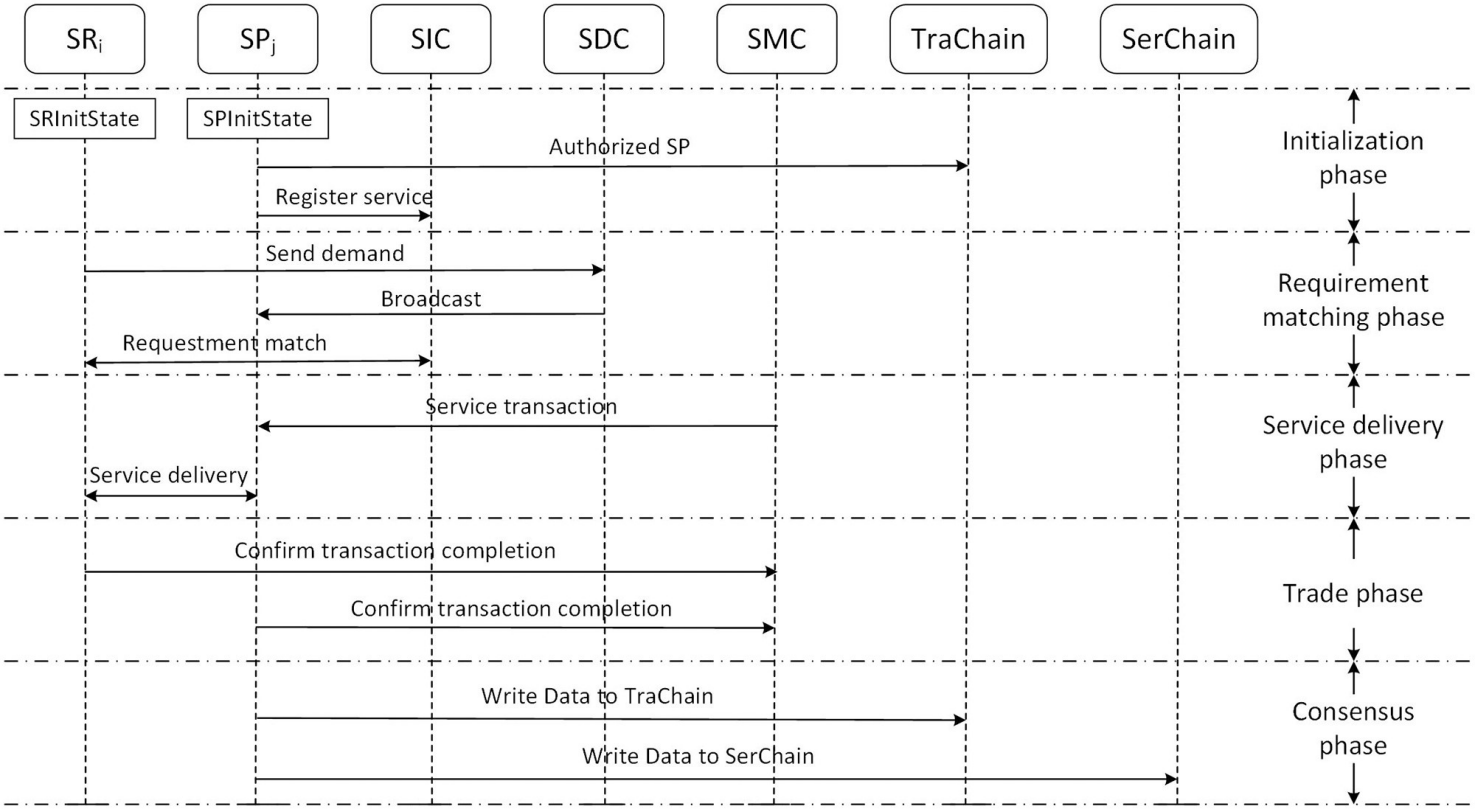
The overall interaction process of the system.

In this section, we will give an overview of the framework. The overall interaction process of the proposed system is presented, which is shown in Fig 2. It contains three important smart contracts mentioned above, a dual-chain architecture, and the interaction process is divided into five stages, namely: initialization phase, requirement matching phase, service delivery phase, trade phase, and consensus phase.

### 3.2 Interaction process

Our main idea is to simulate *SR*_*i*_, *SP*_*j*_, with three smart contracts, and an appropriate consensus mechanism to complete online service transactions effectively and safely. There are five phases as follows.

#### 3.2.1 Initialization phase

In this stage, participants register on STEB and generate their own parameters, such as public-private key pairs. The SP registers service information for sale. Without loss of generality, we assume that there are a total of *n* SRs.

$$SR=\{SR_{1},SR_{2},\ldots ,SR_{n}\}$$ (1)

Specifically, each *SR*_*i*_ generates a ECDSA key pair:

$$\left(PK_{sr_{i}},SK_{sr_{i}}\right)$$ (2)

Its address *Addr*_*i*_ is computed by hashing its public key *PK*_*sri*_. We assume there are a total of *m* SPs:

$$SP=\{SP_{1},SP_{2},\ldots ,SP_{m}\}$$ (3)

Each *SP*_*j*_ generates a ECDSA key pair:

$$\left(PK_{sp_{j}},SK_{sp_{j}}\right)$$ (4)

Its address *Addr*_*j*_ is computed by hashing its public key $PK_{sp_{j}}$. The *SP*_*j*_ register one or more services:

$$S=\{S_{1},S_{2},\ldots ,S_{k}\}$$ (5)

For a new *SP*_*j*_, it should be authorized. For a new service *S*_*r*_, it should be certified. The relation between *SP*_*j*_ and *S*_*r*_ can be represented by *m* × *k* matrix *P* which is represented as

$$\begin{array}{c} P=[\begin{array}{cccc} SP_{1}S_{1} & SP_{1}S_{2} & \ldots & SP_{1}S_{k}\\ SP_{2}S_{1} & SP_{2}S_{2} & \ldots & SP_{2}S_{k}\\ \ldots & \ldots & \ldots & \ldots \\ SP_{m}S_{1} & SP_{m}S_{2} & \ldots & SP_{m}S_{k} \end{array}] \end{array}$$ (6)

All *State*_*id*_ are currently in the states of SRInitState and SPInitState, respectively.

#### 3.2.2 Requirement matching phase

In this section, SR needs to find an appropriate service and its service provider through STEB. We define this process as the requirements matching phase. Algorithm 1 shows more detailed steps as follows. At this step, the smart contract SDC requires SR to send their requirements to it. Specifically, each *SR*_*i*_ computes the hash value:

$$H_{i}=H(SR_{i}||Addr_{i}\left||sid\right)$$ (7)

For each *SR*_*i*_ satisfies the basic condition, it needs to match the correct service and choose the most suitable SP who provides the service. We design the matching function *H* to calculate the matching value, then sort the calculation results in descending order, and return the recommendation list for the SR to choose. We assume a matching function *H* as:

$$H\left(SP_{j}S_{r}\right)=\sum_{j=1}^{m}f\left(S_{r}\right)*w_{j}-p$$ (8)

where, *f*(*S*_*r*_) represents the value of *S*_*r*_, and *w*_*j*_ is the weight, *p* is the punishment factor.

**Algorithm 1** Requirement matching phase

1: require SRInitState, SPInitState, ServiceInitState

2: *SR*_*i*_ request service

3: **for** *r* = 1 to *k* **do**

4:  S[r] = match(*SR*_*i*_, *S*_*r*_)

5: **end for**

6: S[k] result in descending order

7: **for** *j* = 1 to *m* **do**

8:  Calculate *H*(*SP*_*j*_*S*_*k*_)

9:  **if** *H*(*SP*_*j*_*S*_*k*_)> = 0 **then**

10:   *List*[*j*] = *H*(*SP*_*j*_*S*_*k*_)

11:  **else**

12:   *List*[*j*] = 0

13:  **end if**

14: **end for**

15: Sort List in a descending order

16: return List

#### 3.2.3 Service delivery phase

**Fig 3:**
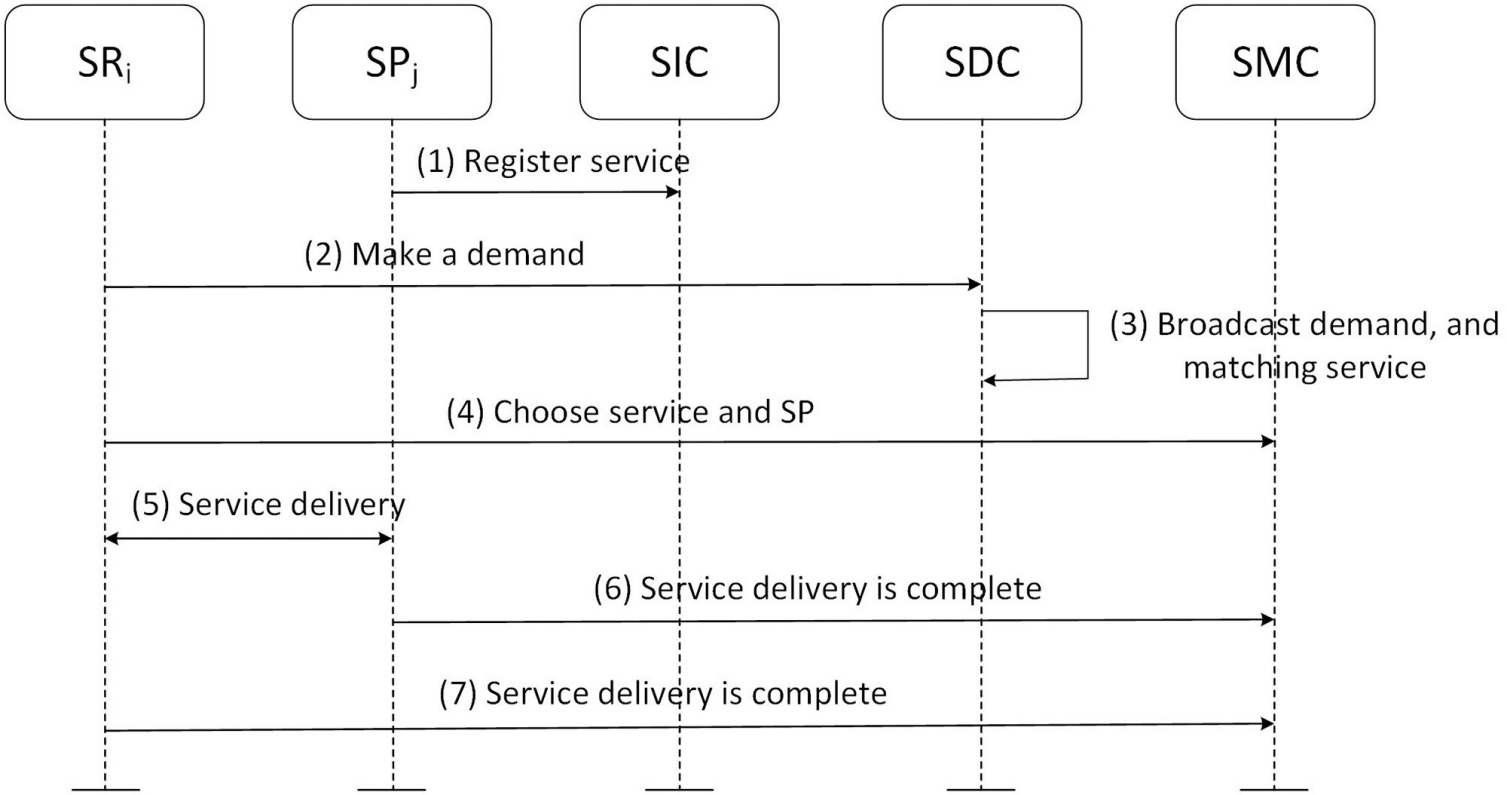
Service delivery phase.

In this section, the SP delivers service to the SR’s business. The completion of the transaction is negotiated by both parties. When requirement matching is finished, the SR chooses the suitable service from the List. Then the selected SP delivers the specified service to SR. The service delivery process is negotiated by SP and SR. It can be done online or offline. After that, SR checks whether the service delivery satisfies their requirements. The process of service delivery phase is shown in Fig 3.

#### 3.2.4 Trade phase

In this phase, the smart contract automatically executes the transaction. Grant the user the evaluation authority of the service. If there is no dispute, transfer the deposit of the corresponding user to the provider. The smart contract requires the SR to pay the service to SP. If the SR rejects to pay, SP will get its payments as soon as time is reached.

#### 3.2.5 Consensus phase

**Fig 4:**
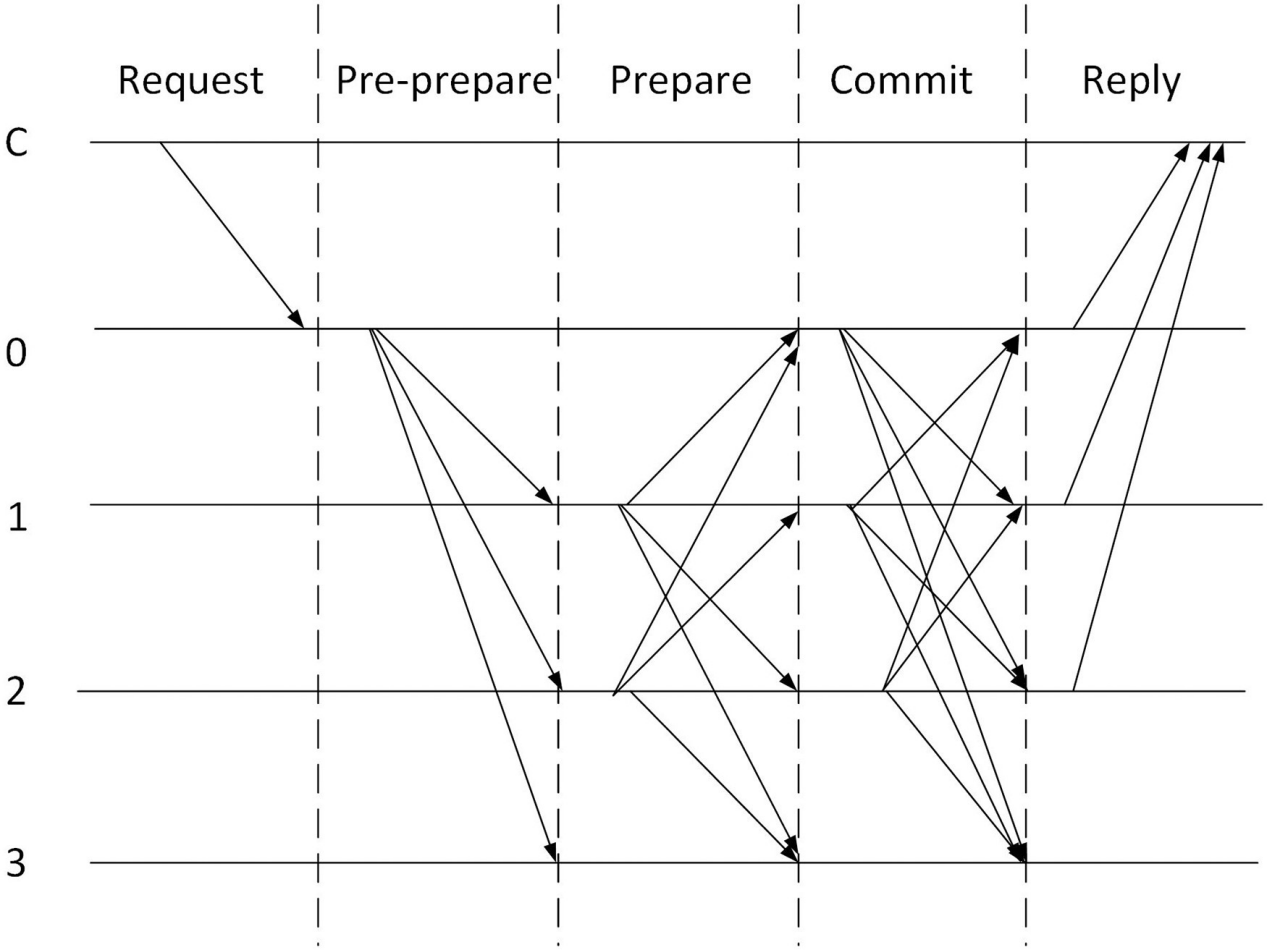
Flowchart of PBFT algorithm.

Since blockchain is a P2P network, each node may receive different transactions at a certain time. The consensus mechanism needs to determine when and which node to add new blocks to the blockchain for the transactions it receives. Since STEB is a consortium blockchain, we used the PBFT as the consensus of the blockchain. The PBFT model provides a Byzantine fault-tolerant algorithm that is resistant to malicious attacks and software errors caused by faulty and malicious nodes. In this paper, only authorized SP nodes participate in the consensus, and the set of SP nodes constitutes the consensus group. Nodes in the consensus group are sequentially ordered, the one node is selected as the primary (so-called leader node), and the others considered as secondary (so-called backup nodes). Every round of PBFT has three phases: pre-prepare phase, prepare phase, and commit phase, as shown in Fig 4.

### 3.3 Dual-chain architecture and hierarchical encryption

There are two chains in STEB, namely TraChain and SerChain. TraChain and SerChain have different permissions, allowing different levels of sensitivity to view user privacy data. This paper adopts hierarchical encryption method to protect the security of transactions and the privacy of users. Divide data into two parts: privacy data and public data:

- Private data *Data*_*pri*_
  Including the user’s personal private information, transaction account address, and other private information.
- Public data *Data*_*pub*_
  Including basic service information, service transaction data, transaction quantity, and other publicly available information.

SerChain nodes are only allowed to view *Data*_*pub*_, and TraChain nodes are allowed to view *Data*_*pub*_ and *Data*_*pri*_. The hierarchical encryption process is as follows:

- The first layer of encryption
  1. Generate public-private key pair for TraChain node A:
    $$\left(PK_{MC_{A}}/SK_{MC_{A}}\right)$$ (9)
  2. Encrypt *Data*_*pri*_ with a symmetric key *Key*_*DES*1_ to form a ciphertext $CText_{Data_{pri}}$.
    $$Data_{pri}\overset{Key_{DES1}}{\to }CText_{Data_{pri}}$$ (10)
  3. Encrypt the symmetric key *Key*_*DES*1_ with public key $PK_{MC_{A}}$ to form a cipherkey $Key_{DES1}-PK_{MC_{A}}$.
    $$Key_{DES1}\overset{PK_{MC_{A}}}{\to }\left(Key_{DES1}-PK_{MC_{A}}\right)$$ (11)
  4. Store Ciphertext $CText_{Data_{pri}}$ and cipherkey $Key_{DES1}-PK_{MC_{A}}$ on the blockchain.
- The second layer of encryption
  1. Generate public-private key pair for SerChain node B:
    $$\left(PK_{MC_{B}}/SK_{MC_{B}}\right)$$ (12)
  2. Encrypt $CText_{Data_{pri}}$ and *Data*_*pub*_ with a symmetric key *Key*_*DES*2_ to form ciphertext $CCText_{Data_{pri}}$ and $CText_{Data_{pub}}$, respectively.
    $$CText_{Data_{pri}}\overset{Key_{DES2}}{\to }CCText_{Data_{pri}}$$ (13)
    $$Data_{pub}\overset{Key_{DES2}}{\to }CText_{Data_{pub}}$$ (14)
  3. Encrypt the symmetric key *Key*_*DES*2_ with public key $PK_{MC_{A}}$ and $PK_{MC_{B}}$ to form a cipherkey $Key_{DES2}-PK_{MC_{A}}$ and $Key_{DES2}-PK_{MC_{B}}$, respectively.
    $$Key_{DES2}\overset{PK_{MC_{A}}}{\to }\left(Key_{DES2}-PK_{MC_{A}}\right)$$ (15)
    $$Key_{DES2}\overset{PK_{MC_{B}}}{\to }\left(Key_{DES2}-PK_{MC_{B}}\right)$$ (16)
  4. Store Ciphertext $CCText_{Data_{pri}}$, $CText_{Data_{pub}}$, $Key_{DES2}-PK_{MC_{A}}$, and $Key_{DES2}-PK_{MC_{B}}$ on the blockchain. The hierarchical decryption process is as follows:
- The first layer of decryption
  1. SerChain node C makes an acquisition request, and adds a timestamp, the format is as follows:
    $$Request_{C}=(Data_{C}\left||Timestamp\right)$$ (17)
  2. Identity verification of C is performed just once, when verified, the request data $CText_{Data_{C}}$ has been reply to C.
    $$Reply_{C}=(CText_{Data_{C}}||Timestamp^{\prime })$$ (18)
  3. C read $Key_{DES2}-PK_{MC_{B}}$ from blockchain, decrypt it with the private key, hence getting the key *Key*_*DES*2_. Then decrypt $CText_{Data_{C}}$ with the symmetric key *Key*_*DES*2_, hence getting the data $CText_{Data_{pri}}$ and *Data*_*pub*_.
    $$\left(Key_{DES2}-PK_{MC_{B}}\right)\overset{SK_{MC_{B}}}{\to }Key_{DES2}$$ (19)
    $$CText_{Data_{C}}\overset{Key_{DES2}}{\to }\left(Data_{pub},CText_{Data_{pri}}\right)$$ (20)
    At this moment, SerChain node C can read *Data*_*pub*_, but $CText_{Data_{pri}}$ is a ciphertext and cannot be read directly. If there is an TraChain node D, which has greater permission, it needs to enter second layer of decryption.
- The second layer of decryption
  1. TraChain node D makes an acquisition request, and adds a timestamp, the format is as follows:
    $$Request_{D}=(Data_{D}\left||Timestamp\right)$$ (21)
  2. Identity verification of D is performed, when verified, the requested data $CText_{Data_{D}}$ has been replied to D.
    $$Reply_{D}=(CText_{Data_{D}}||Timestamp^{\prime })$$ (22)
  3. After the first layer of decryption, D gets the data as follows:
    $$CText_{Data_{D}}\overset{Key_{DES2}}{\to }\left(Data_{pub},CText_{Data_{pri}}\right)$$ (23)
  4. D get $Key_{DES1}-PK_{MC_{A}}$ from blockchain, decrypt it with the private key, hence getting the key *Key*_*DES*1_. Then decrypt $CText_{Data_{pri}}$ with the symmetric key *Key*_*DES*1_, hence getting the data *Data*_*pri*_.
    $$CText_{Data_{pri}}\overset{Key_{DES1}}{\to }\left(Data_{pri}\right)$$ (24)
    After the second layer of decryption, TraChain node D can read *Data*_*pri*_ and *Data*_*pub*_ both.

## 4 Security and performance analysis

In this section, we experiment to validate the security analysis and simulation results of STEB.

### 4.1 Security analysis

STEB has defense ability against many traditional security attacks through dual-blockchain and standard cryptographic primitives including asymmetric and symmetric key-based encryption. The following blockchain-related security benefits can be satisfied in the proposed consortium blockchain enabled multi-party service trading scheme.

#### 4.1.1 Privacy protection

Due to the dual-chain mechanism, the price and quantity of the sales service are only submitted to STEB without private information during the service transaction process. The hierarchical data encryption scheme authorizes users to have different access rights, thereby protecting their identity privacy and account information security. Due to the encryption and authentication mechanisms, the adversary cannot open the encrypted messages by launching brute force cryptanalytic attacks.

#### 4.1.2 Auditability

Blockchain is a distributed ledger that records all operations and initiators in the process of service transactions, and cannot be tampered with. The interaction between sellers and buyers is verified and stored in a distributed P2P manner, without the need for the only trusted third party, making the system robust and scalable. Therefore, once the service transaction is committed, the SR cannot deny the transaction it initiated, nor the SP to deny the operation it initiated. Since a digital signature is only generated by a specific signer, any information with a digital signature can be authenticated and verified whether the signer is the sender or not. All entities verify the digital signature during the communication. Without the private key of the signer, any entity cannot counterfeit the digital signatures of other entities. Denials or illegal behaviors can be audited, analyzed, tracked and traced through digital signatures.

#### 4.1.3 Integrity

Consortium blockchains are supervised, which means that entities participating in transactions can also be supervised. If one message is modified, the receivers can discover it during verification, which guarantees the integrity. While smart contracts SIC, SRC, and SMC have been deployed on the blockchain, and cannot be changed once deployed. All information exchanged between nodes cannot be tampered with, which means that all service transaction information and certification will not be tampered with. Therefore, when a dispute occurs in service transaction, the SR cannot deny his/her behavior, nor can the SP deny his/her behavior. Thus, our scheme could provide the security of transaction information and ensures the integrity of transaction information.

### 4.2 Performance analysis

We implement our prototype model to the Hyperledger Fabric [16] blockchain platform to evaluate the performance. Hyperledger Fabric is a modular and extensible open-source system for deploying and operating permissioned blockchains, which has a private and permissioned network and whose modular architecture maximizes blockchain secrecy, flexibility and facilitates decision-making capabilities. Hyperledger caliper, which is a benchmark framework that supports Transaction Latency (TL), Transaction Throughput (TT), and other performance analysis, is used to evaluate the performance of the proposed blockchain-based system. All the experiments were done on CentOS 7.8, 64bit with Intel Core i7, 10750Hz and 8G RAM.

#### 4.2.1 Transaction latency

Transaction latency measures the time for a transaction from the point that it is submitted to the point that it is committed, including the propagation time and any time due to consensus mechanisms. Its calculation formula is as follows:

$$TL=t_{commit}-t_{submit}$$ (25)

where, *t*_*commit*_ represents the time when the transaction is committed, *t*_*submit*_ represents the time to send a transaction proposal.

The proposed scheme is deployed on a Fabric blockchain simulation platform based on Docker. It contains five peer nodes and one ordering node. The endorsement policy is Peer 0: org 1 to Peer 0: org 5, which are both required to sign the transaction to determine the validity of the transaction.

**Fig 5:**
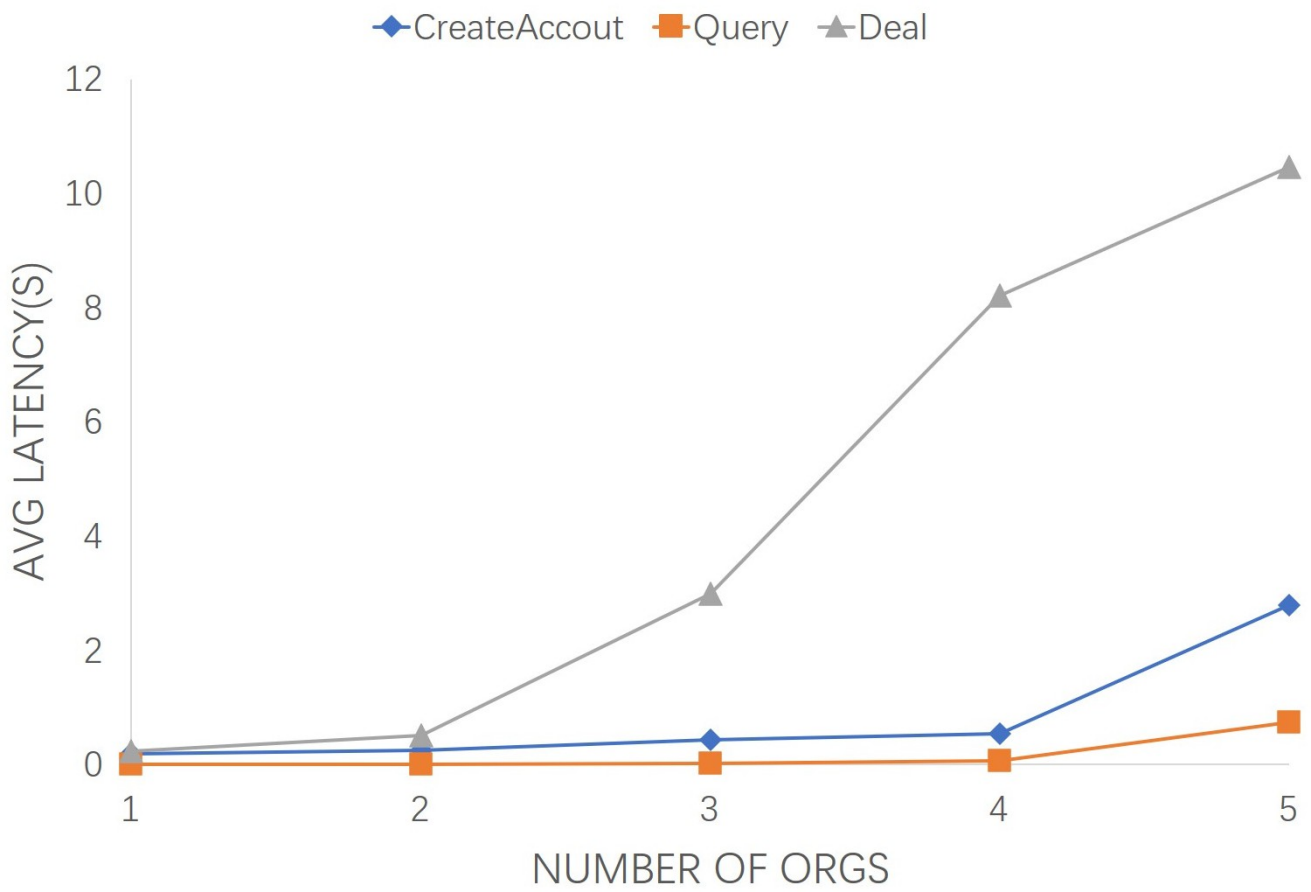
Transaction latency of the number from 1 to 5 of orgs.

Fig 5 describes the effect of the number from 1 to 5 of orgs on the average TL. It can be observed that the average latency of “CreateAccout” and “Query” increase slightly as the number of orgs grow. This means that the increase in orgs have little effect on them. The average latency of “Deal” increases linearly with the growth of org number. The average TL of “Deal” for the defined 1 to 5 org is 0.172 seconds (s). Likewise, in the case of 5 orgs, the average TL of “Deal” is increased from 0.24 to 10.46 seconds. The increase of endorsing nodes has a linearly related effect on transaction latency.

**Fig 6:**
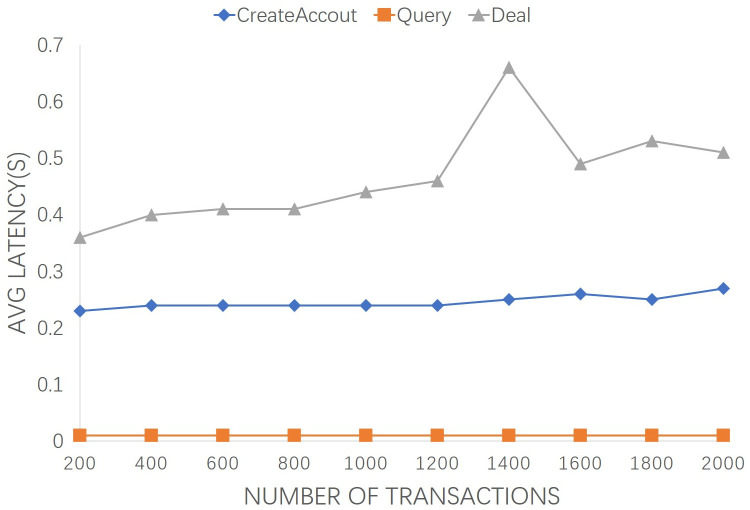
Transaction latency of the involved transactions from 200 to 2000.

According to existing blockchain-based research, the number of concurrent transactions is defined in the range of 200 to 2000 to evaluate transaction execution. Fig 6 describes the effect of the number of concurrent transactions on the average TL in the case of 2 peer nodes and 1 ordering node. We provide the number of transactions from 200 to 2000 to the blockchain system to evaluate the results of executing the transactions in terms of “CreateAccout”, “Query” and “Deal” latency. The average “CreateAccout” TL of the executing transactions for all 200, 1000, and 2000 is 0.23, 0.24, and 0.27 seconds, respectively. The average “Query” TL of the executing transactions for 200, 1000, and 2000 are 0.01, 0.01, and 0.01 seconds(s), respectively. Whereas, the average “Deal” TL of the executing transactions for 200, 1000, and 2000 are 0.36, 0.44, and 0.51 seconds(s), respectively. It can be observed that the average latency increases slightly with the growth of transaction number. The average latency fluctuates slightly at number of 1400 due to the test environment, but generally maintains a stable growth trend.

#### 4.2.2 Transaction throughput

Transaction throughput represents the rate at which valid transactions are committed by the blockchain in a defined time period. The throughput of a given blockchain is defined by transactions per second (TPS). Its calculation formula is as follows:

$$TT=\frac{\;\text{transactions}\;}{\Delta t}$$ (26)

where, *transactions* is the number of transactions that written to the blockchain.

**Fig 7:**
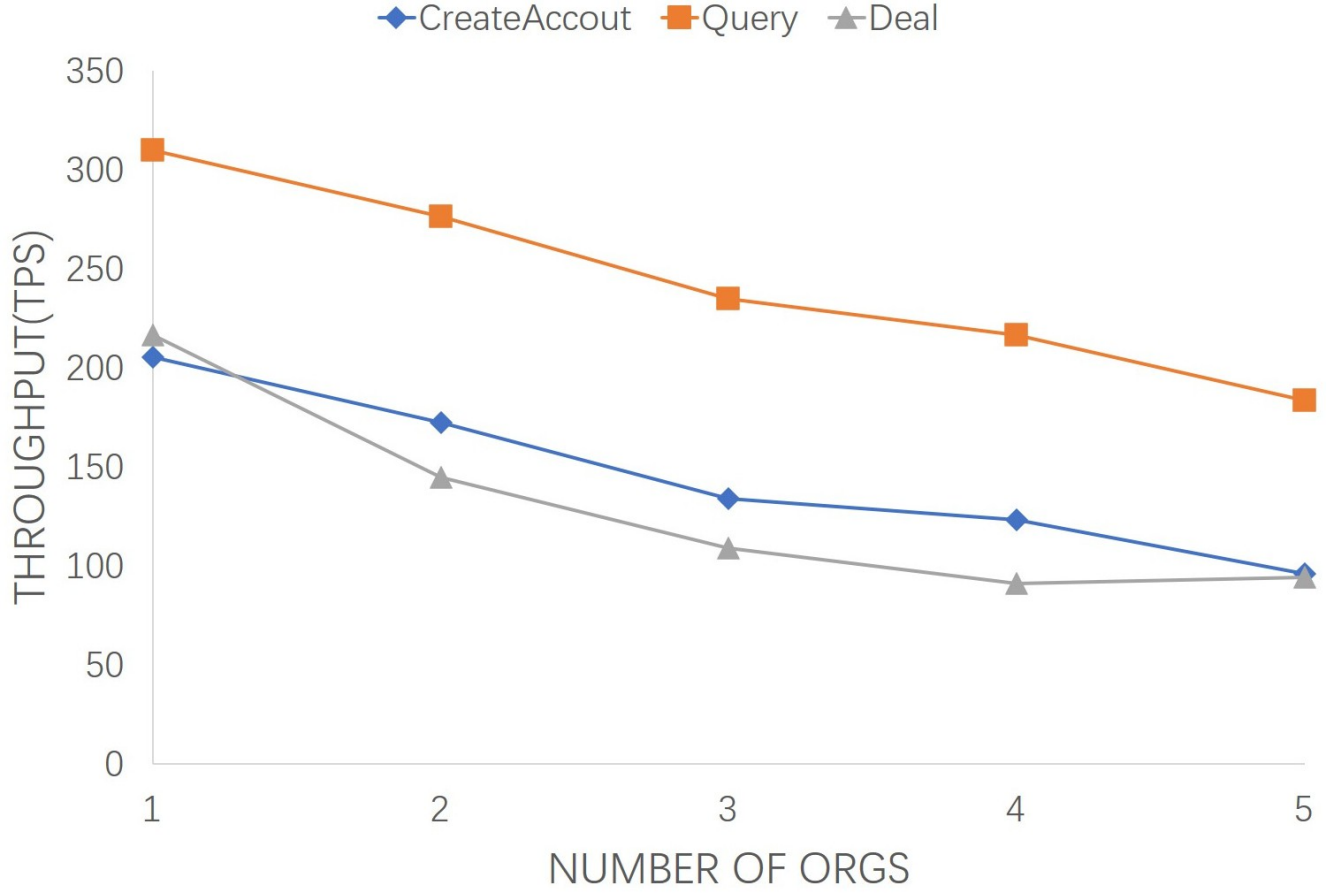
Transaction throughputs of the number from 1 to 5 of orgs.

Similarly, in Fig 7, the effect of the number from 1 to 5 of orgs on transaction throughput are analyzed based on average performance of the proposed system. It can be observed that the average transaction throughput of “CreateAccout”, “Query” and “Deal” decrease linearly with the growth of org number. The increase of endorsing nodes has a linearly related effect on transaction throughput. When the number of endorsing nodes increases, more nodes participate in the consensus process. It takes more time to complete transaction query, commit, deal, and consensus, but the overall trend remains stable approximately.

**Fig 8:**
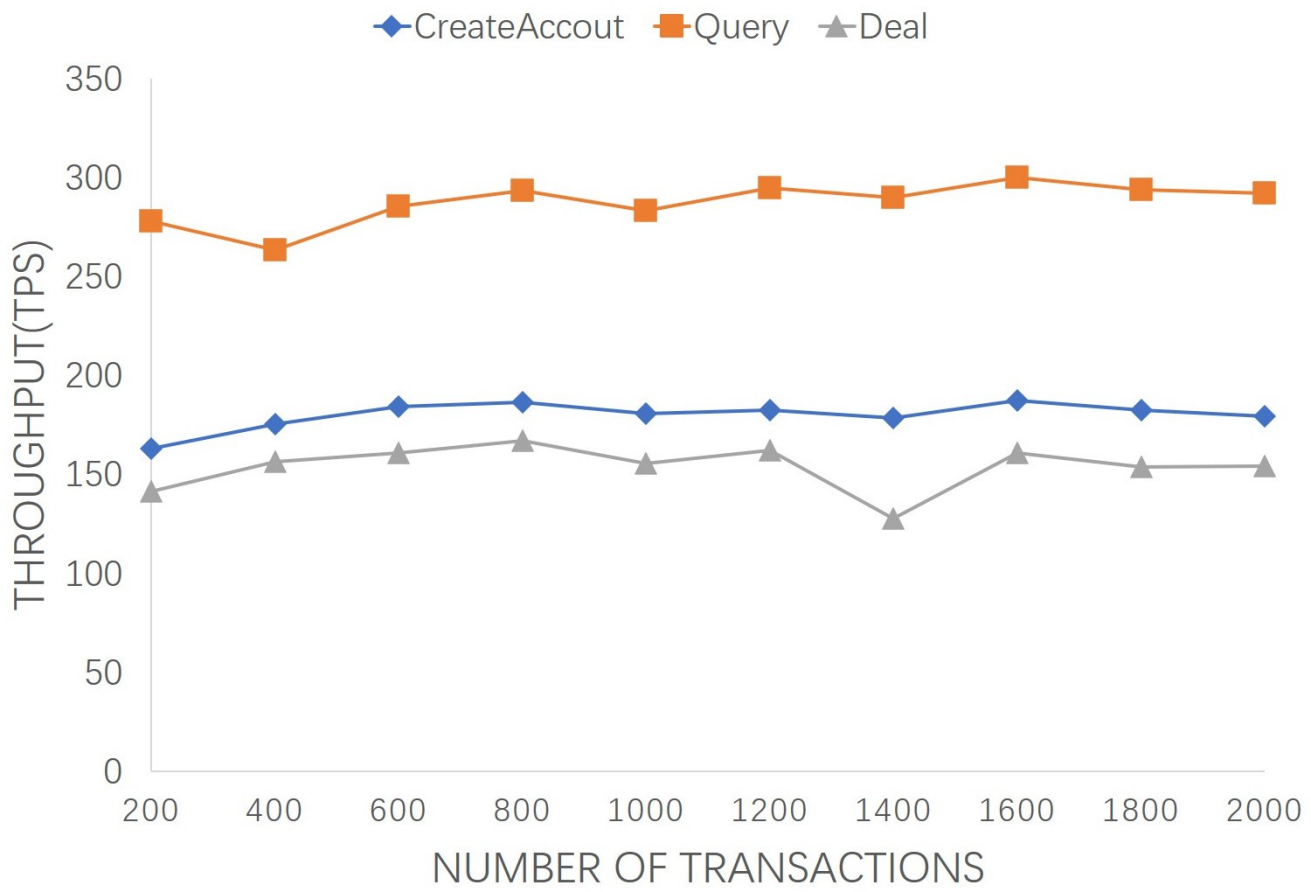
Transaction throughputs of the involved transactions from 200 to 2000.

In Fig 8, the effect of the number of concurrent transactions on transaction throughput in the case of 2 peer nodes and 1 ordering node are analyzed based on average performance of the proposed system. The average “CreateAccout” TT of the executing transactions for 200, 1000, and 2000 are 162.9, 180.8, and 179.4 TPS, respectively. The average “Query” TL of the executing transactions for 200, 1000, and 2000 are 277.8, 283.1, and 292.3 TPS, respectively. Whereas, the average “Deal” TL of the executing transactions for 200, 1000, and 2000 are 141.6, 155.7, and 154.3 TPS, respectively. It can be observed that during the process of increasing the number of concurrent transactions from 200 to 2000, the throughput fluctuated slightly under the influence of the test environment, but the overall trend has remained stable.

In practice, both latency and throughput factors are need to be comprehensively considered. Long-term transaction response will affect system response time and users’ experience. Small throughput will cause a large number of transactions to be queued. From Figs 5 and 7, the average latency and transaction throughput performance remain stable as the number of org increases. Similarly, from Figs 6 and 8, with the growth of transaction numbers, the TT of the proposed system is increased, but it also increased TL due to transmitting an increasing number of consensus messages. The increase curve is stable and linear, indicating the stability and efficiency of the proposed scheme.

## 5 Conclusion

In this paper, we propose a secure service trading ecosystem based on blockchain that provides a secure and trusted transaction environment for multi-participants. A new set of smart contracts to ensure safe transactions is described for the entire service trading. A dual-chain architecture and a hierarchical encryption scheme of the data on the chain are proposed to protect the integrity of transaction data and fine-grained privacy protection of users. Security analysis and performance evaluation on the Hyperledger Fabric blockchain platform confirms the effectiveness and feasibility, and simulation results indicate that the proposed STEB can achieve more efficient contract execution and enhance service transaction privacy.
